# Reducing microbial ureolytic activity in the rumen by immunization against urease therein

**DOI:** 10.1186/s12917-015-0409-6

**Published:** 2015-04-14

**Authors:** Shengguo Zhao, Jiaqi Wang, Nan Zheng, Dengpan Bu, Peng Sun, Zhongtang Yu

**Affiliations:** Ministry of Agriculture Laboratory of Quality & Safety Risk Assessment for Dairy Products (Beijing), Institute of Animal Science, Chinese Academy of Agricultural Sciences, No. 2 Yuanyingyuan West Road, Beijing, 100193 PR China; State Key Laboratory of Animal Nutrition, Institute of Animal Science, Chinese Academy of Agricultural Sciences, No. 2 Yuanyingyuan West Road, Beijing, 100193 PR China; Department of Animal Sciences, The Ohio State University, Columbus, OH 43210 USA

**Keywords:** Immunization, Rumen, Urease, Ureolytic activity

## Abstract

**Background:**

Ureolytic activity of rumen bacteria leads to rapid urea conversion to ammonia in the rumen of dairy cows, resulting possible toxicity, excessive ammonia excretion to the environment, and poor nitrogen utilization. The present study investigated immunization of dairy cows against urease in the rumen as an approach to mitigate bacterial ureolytic activity therein.

**Results:**

Most alpha subunit of rumen urease (UreC) proteins shared very similar amino acid sequences, which were also highly similar to that of *H. pylori*. Anti-urease titers in the serum and the saliva of the immunized cows were evaluated following repeated immunization with the UreC of *H. pylori* as the vaccine. After the fourth booster, the vaccinated cows had a significantly reduced urease activity (by 17%) in the rumen than the control cows that were mock immunized cows. The anti-urease antibody significantly reduced ureolysis and corresponding ammonia formation in rumen fluid *in vitro*. Western blotting revealed that the *H. pylori* UreC had high immunological homology with the UreC from rumen bacteria.

**Conclusions:**

Vaccine developed based on UreC of *H. pylori* can be a useful approach to decrease bacterial ureolysis in the rumen.

**Electronic supplementary material:**

The online version of this article (doi:10.1186/s12917-015-0409-6) contains supplementary material, which is available to authorized users.

## Background

Ruminal microbial urease plays an important role in the nitrogen metabolism in ruminants such as cattle and sheep. The urea from diet or recycled from blood to rumen is hydrolyzed by urease to ammonia, the major source of nitrogen for many ruminal bacteria including several known cellulolytic bacteria [1]. The traditional recommendation for urea feeding is less than 1% of the concentrate portion of the diet, approximately 135 g/cow daily [2]. Between 40 and 80% of the urea-N synthesized by the liver also return to the rumen and gut, where 35 to 55% of this N is used in microbial anabolism in both cattle and sheep [3]. However, the rate of urea hydrolysis (ureolysis) is about four fold greater than that of ammonia assimilation, resulting in ammonia accumulation, which can lead to toxicity, excessive ammonia excretion to the environment, and poor N utilization when diets contain a high urea content [2,4]. To alleviate this problem, different urease inhibitors have been evaluated to reduced ureolytic activity, including acetohydroxamic acid, phenyl phosphorodiamidate, N-(n-butyl) thiophosphoric triamide, boric acid, and bismuth compounds, to slow down production of ammonia in the rumen [5]. However, their efficacy decreases over time due to microbial adaptation [6], and some of these compounds pose potential risk animal and human health, precluding their use in production.

Recent studies have shown that immunization is a potential approach to mitigate methane emissions [7-9], lactic acidosis [10], and to decrease protozoal population [11] in the rumen. We hypothesize that immunization against rumen urease can be an effective approach to slow down ureolysis in the rumen. Bacterial urease consists of two (alpha and beta) or three (alpha, beta and gamma) structural subunits. The alpha subunit (UreC) contains a urea binding site and a catalytic site. The *ure*C gene has been used as a gene marker to examine UreC diversity in bacterial communities because it is quite conserved among different bacterial species [12]. In one study, the UreC of jack bean urease has been tried as an antigen to immunize sheep to inhibit rumen ureolysis in sheep rumen [13]; however, no obvious anti-urease activity was achieved probably because of low immunological homology between jack bean urease and rumen bacterial urease. The objectives of the present study were to examine the diversity of UreC in the rumen, identify an antigen that has high immunological homology with rumen UreC, develop an anti-urease vaccine from bacterial UreC, and evaluate anti-urease immunization as an approach to decrease ruminal ureolysis.

## Results

### Diversity of rumen bacterial urease gene

**Figure 1:**
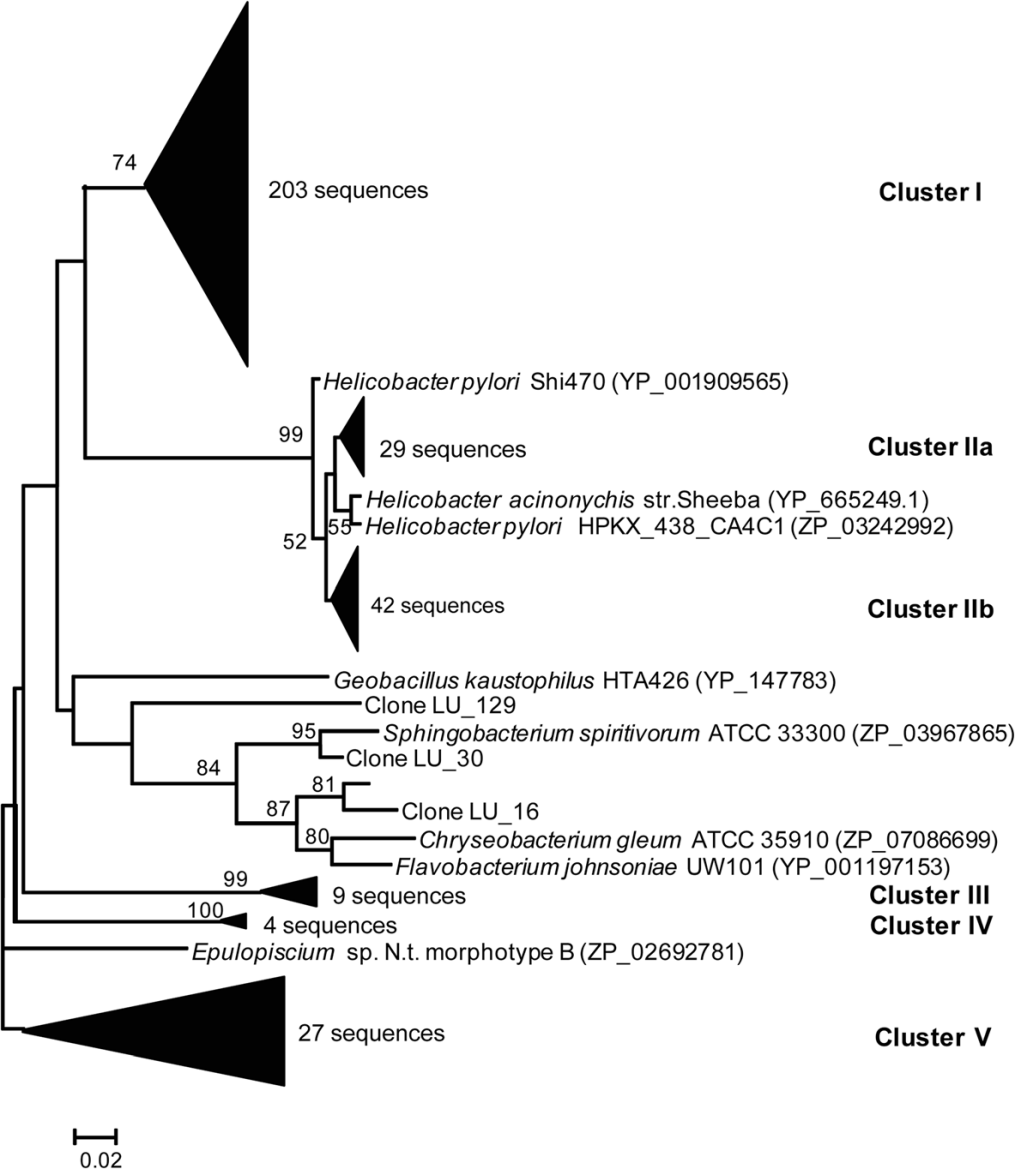
A neighbor-joining tree of the UreC sequences recovered from rumen digesta. The consensus tree was constructed from amino acid sequences inferred from the *ure*C sequences recovered from rumen and known bacterial species. Bootstrap values were calculated from 1,000 trees. Only bootstrapping values greater than 50% are shown.

The *ure*C diversity in the rumen was examined by cloning and sequencing of *ure*C genes using degenerate primers. In total, 317 *ure*C sequences were obtained from the microbial DNA of rumen digesta of Chinese Holstein cows. Phylogenetic analysis revealed five *ure*C clusters (Figure 1). Cluster I contained 203 (64% of total sequences) of the *ure*C sequences, and it was about 84% identical (based on amino acid sequence) to the *ure*C gene of *Helicobacter pylori* (*H. pylori)*. Clusters IIa and IIb represented 29 (9%) and 42 (13%) of the *ure*C sequences, respectively, and both were closely related (98-100% aa sequence identity) to the *ure*C of *H. pylori*. Clusters III and IV, each of which contained a small number of *ure*C sequences, and cluster V, represent the rest of the *ure*C sequences, had no match with any known *ure*C sequences.

### Immunological homology between purified rumen urease and ***H. pylori*** urease

**Figure 2:**
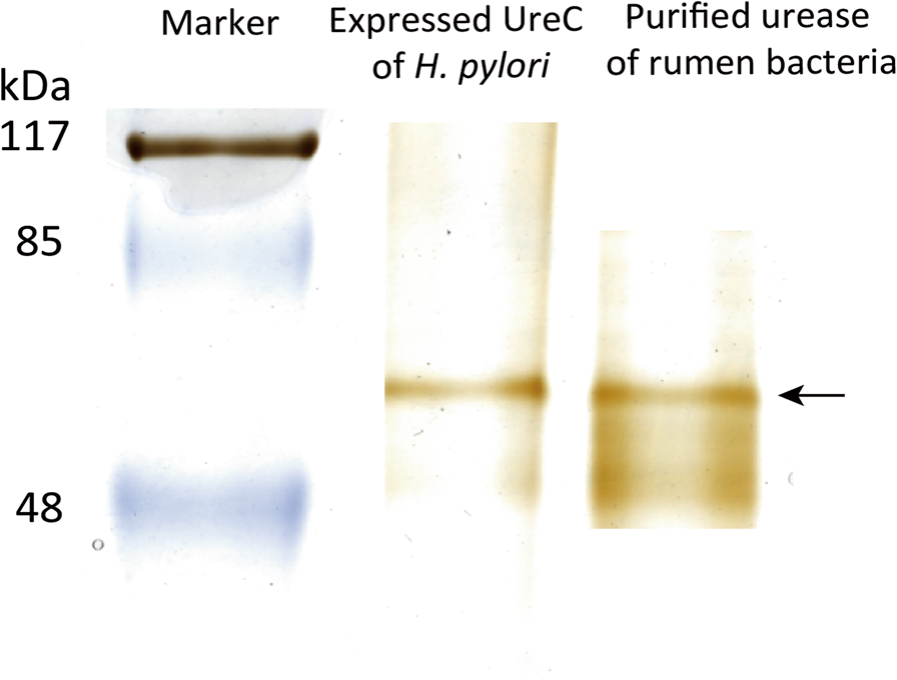
Western blot of urease purified from the rumen of dairy cows using anti-urease serum collected from cows immunized with overexpressed UreC of *H. pylori* **.** The UreC band was indicated by arrow.

Immunological homology between urease purified from the rumen and the *H. pylori* urease was evaluated using Western blotting. Urease protein with an activity of 542 U was purified from rumen bacteria by anion exchange chromatography. Western blotting of the purified urease using anti-urease serum from the cows immunized with overexpressed UreC of *H. pylori* identified the positive band of expected molecular weight (Figure 2), indicating a high immunological homology between the overexpressed UreC of *H. pylori* and the urease purified from the rumen bacteria.

### Vaccine and specific antibody titers

The above *ure*C diversity data showed that the majority (86%) of the UreC in the rumen share high homology (84-100% aa sequence identity) with the UreC of *H. pylori*. The urease of *H. pylori* also share high immunological homology with the urease of rumen bacteria. Therefore, *H. pylori* UreC was selected as the antigen to elicit immunization against urease in the rumen of dairy cows. Another reason to choose the UreC of *H. pylori* was the availability of full-length sequence of its *ure*C gene so that this UreC protein can be overexpressed in *E. coli*. The UreC of *H. pylori* was successfully expressed in *E.coli* BL21(DE3) following induction with IPTG. The molecular weight of the expressed UreC was about 66 kDa, consistent with the molecular mass predicted from the UreC sequence (see Additional file 1). About 20 mg purified UreC was obtained. The expressed UreC protein, together with Freund’s adjuvant, was used as the vaccine to immunize the dairy cows.

**Figure 3:**
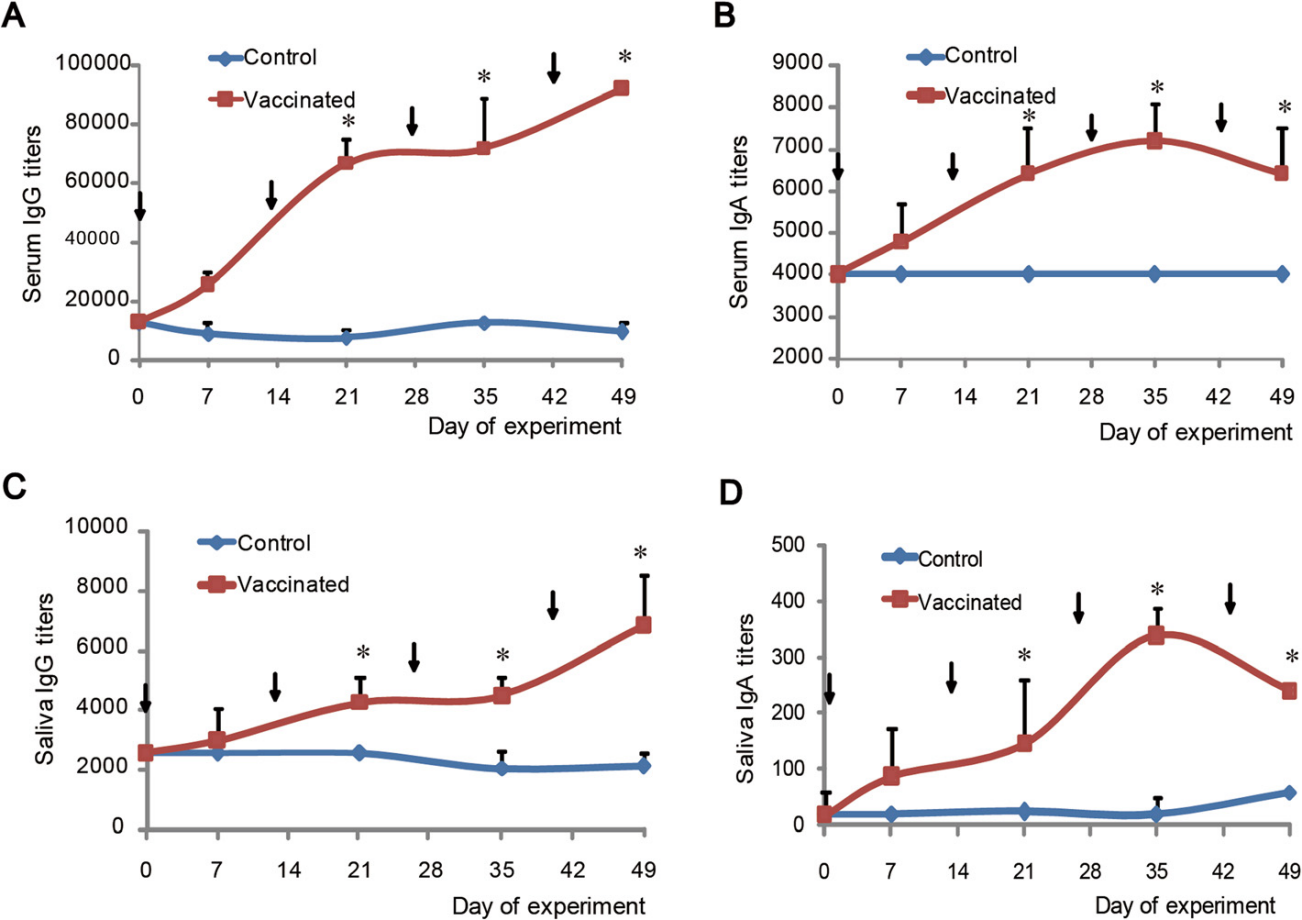
Titers of IgG (**A** and **C**) and IgA (**B** and **D**) in the serum (**A** and **B**) and the saliva (**C** and **D**) of cows. Arrow indicates days of vaccinations. Values are means (n = 4), with error bars representing standard deviation. The asterisks (*) indicate significant (P < 0.05) difference between the control group and the vaccinated group at the same days.

After the immunization with *H. pylori* UreC, no apparent adverse effect was seen on health, milk production, or digestion of dry matter and crude protein (data not shown). Low titers of anti-urease antibody were detected in the serum and the saliva samples from the control group from day 0 (prior to mock immunization) to day 49 (Figure 3). Compared to the control group, the vaccinated group had higher (P < 0.01) serum titers of both IgG and IgA from day 7 onward, while higher (P < 0.01) saliva titers of IgG and IgA were noted from days 21 and 7 onward, respectively. The IgA titer peaked at day 35 in both the serum and the saliva, but the IgG titers peaked later at day 49. The variation of both IgA and IgG titers had similar trends in the serum and the saliva. The highest titers of both IgG and IgA in the serum were 13- and 20-fold greater, respectively, than those noted for the saliva.

### Urease activity and rumen fermentation after immunization

**Figure 4:**
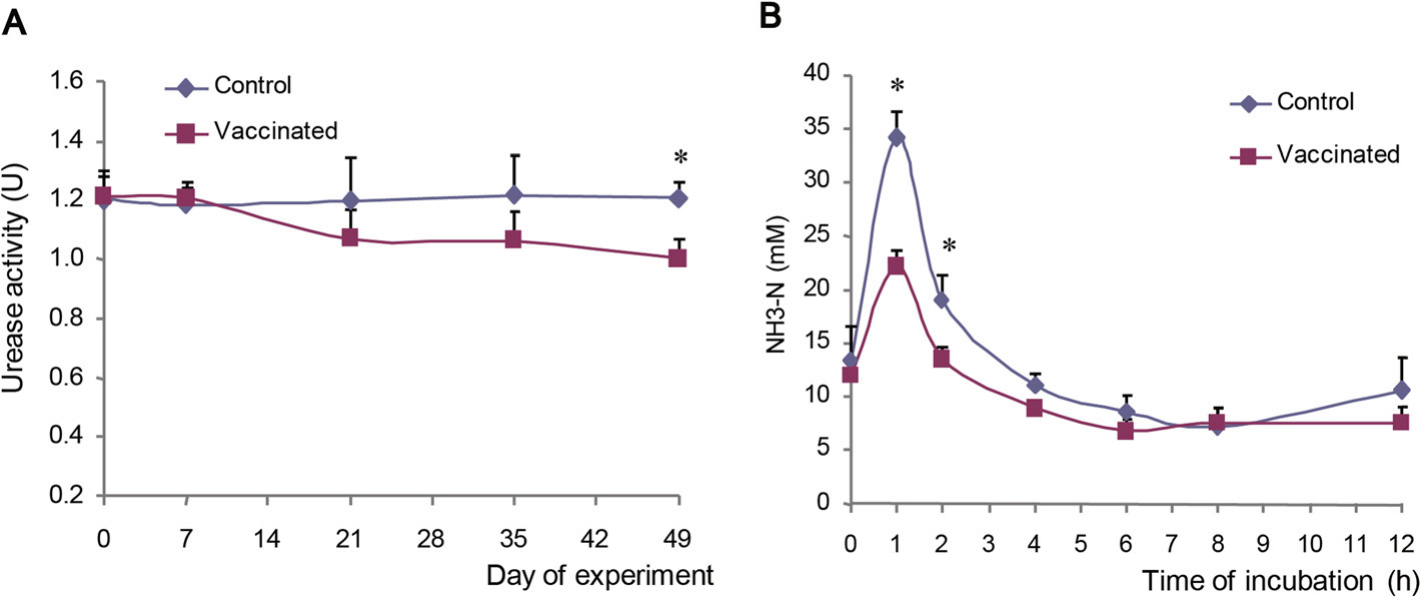
Urease activity in the rumen after immunization (**A**) and ammonia concentration variation after urea was infused into the rumen (**B**). Values are means (n = 4), with error bars representing standard deviation. The asterisks (*) indicate significant (P < 0.05) difference between the control group and the vaccinated group at the time points.

The effect of immunization against urease was assessed by analyzing rumen fermentation characteristic and ureolysis in the rumen of the vaccinated cows. No significant difference in rumen urease activity was seen between the control and the vaccinated groups from days 0 to 35 (before the 3^rd^ booster) (Figure 4A). At day 49 (two weeks after the third booster), however, urease activity in the vaccinated group was 17% lower (P < 0.01) than that in the control group. Rumen pH and volatile fatty acid (VFA) concentration were not affected by the immunization (see Additional file 2). After direct infusion of urea into the rumen at day 56, ammonia concentration in the rumen ascended during the first hour and then descended to the pre-infusion level (Figure 4B). Compared to the control group, the vaccinated group had lower (P < 0.01) ammonia concentration at 1 and 2 h post infusion, but not thereafter.

### Inhibition of urea hydrolysis by anti-urease serum *in vitro*

**Figure 5:**
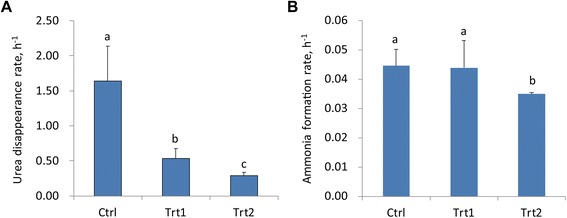
Effect of addition of anti-urease serum to fresh rumen fluid on the rate of urea disappearance (**A**) and corresponding ammonia formation (**B**) i*n vitro*. Values are means (n = 3), with error bars representing standard deviation. The different alphabets above error bars indicate significant (P < 0.05) difference between treatments.

The ability of anti-urease antibodies to decrease ureolysis by rumen microbes was evaluated using fresh rumen fluid *in vitro*. Compared to the control, the addition of serum anti-urease antibody from the vaccinated cows significantly reduced the rate of urea disappearance and corresponding ammonia formation (Figure 5). Urea was completely hydrolyzed within 4 h of the incubation in the absence of the anti-urease serum; however, urea disappearance was slowed down in the presence of the anti-urease serum. Concomitantly, increase in ammonia concentration was reduced within 12 h after the anti-urease serum addition.

## Discussion

Most of the ureases in the rumen are produced by bacteria, but little is known about the diversity of the urease-producing bacteria because only 6.5% of the rumen bacteria have been cultured or characterized [14,15]. The urease genes carried by rumen ureolytic bacteria have not been systematically examined. In a study conducted in 1970′s, Cook et al. [16] found that urease from rumen bacteria, such as *Staphylococcus* spp., was either intracellular or bound to the surface of the cell wall. The diversity of ureolytic bacteria and their urease gene have to be identified before highly effective urease vaccine can be developed. The present study is the first study that examined the diversity of *ure*C genes in the rumen of dairy cows using a cultivation-independent approach. About 86% of the recovered UreC sequences shared high sequence identity (84-100%) to that of *H. pylori*; however, interestingly, none of the recovered UreC sequences matched that previously recovered from any cultured rumen microbes. The UreC from *H. pylori* has been verified to have high immunogenicity in mice and human [17]. In the present study, overexpressed UreC of *H. pylori* was shown to be highly immunologically homologous to rumen UreC of dairy cows. The observed inhibition to urease activity and corresponding decrease in ammonia accumulation suggest that specific antibodies against rumen bacterial urease were produced by the dairy cows vaccinated with the overexpressed UreC of *H. pylori*.

As demonstrated in the present study, urease vaccination did elicit a humoral immune response as indicated by the elevated serum and saliva specific antibody titers observed in the vaccinated cows but not in the control cows. Specific IgG and IgA titers in the serum and the saliva were further increased following booster immunization and peaked after the third and second booster immunization, respectively. The IgG and IgA titers in the serum had high positive correlation with those in the saliva. The specific IgG and IgA could flow into rumen fluid with saliva, because the liquid in the rumen is primarily (>70%) derived from saliva [18]. Although rumen contains proteolytic bacteria, no significant degradation of IgG molecules within the first 4 h of incubation in fresh rumen fluid [19,20]. As such, anti-urease antibodies produced by vaccination can persist long enough in the rumen to bind to urease and reduce ureolytic activity.

Immunization with jack bean urease failed to reduce urease activity or urea kinetics in sheep rumen [13] or produce antibody against the urease of *Helicobacter* in vaccinated mice [21]. This inability is probably attributed to a lack of immunological homology between bacterial urease and jack bean urease. The reduced urease activity by the bovine anti-urease antibody elicited by the UreC of *H. pylori*, both *in vitro* and *in vivo*, clearly indicates that the UreC of *H. pylori* has high immunological homology with rumen bacterial ureases, at least many of them, and can be used as an effective vaccine in cows. The Western blotting further confirmed the immunological homology between the rumen bacterial urease and the *H. pylori* urease. However, given the diverse UreC present in the rumen (Figure 1), a vaccine prepared from a combination of representatives of different rumen UreC clusters may be more effective than UreC of *H. pylori* or single rumen bacterial UreC. Future studies are also needed to identify ureolytic bacteria and their ureases so that anti-urease antibodies with greater efficacy might be developed.

## Conclusions

The alpha subunit of *H. pylori* urease may serve as a vaccine to immunize cows to slow down ureolysis in the rumen. Combined representatives of rumen bacterial UreC may be an even more effective vaccine to improve urea utilization efficiency without the adverse effects associated with chemical urease inhibitors.

## Methods

### Diversity of rumen bacterial urease gene

Rumen digesta samples were collected from four rumen-fistulated Chinese Holstein dairy cows before morning feeding. Total microbial DNA was extracted using the RBB + C method [22]. A degenerate primer set specific for the *ure*C gene (ureC forward:5′-TGGGCCTTAARMTHCAYGARGAYTGGG-3′, and ureC reverse:5′-GTGRTGRCAMACCATNANCATRTC-3′) [23] was used in PCR amplification of the UreC in the rumen samples. A 25 μL PCR reaction contained 2.5 μL PCR buffer (Invitrogen, Carlsbad, CA), 0.75 μL MgCl_2_ (50 mM), 0.5 μL dNTP (10 mM), 1.5 μL each forward and reverse primer (10 μm), 0.3 μL Platinum Taq DNA polymerase (Invitrogen, Carlsbad, CA), 1 μL rumen microbial DNA (~100 ng μL^−1^), and 16.95 μL sterile ddH_2_O. The PCR cycling included 94°C for 5 min; 30 cycles of 94°C for 30 s, 50°C for 30 s, and 72°C for 30 s; 72°C for 15 min; and 10°C for 30 min. The expected PCR amplicons of about 324 bp were visualized on agarose (2%) gel and then purified using a Gel Purification Kit (Qiagen, Valencia, CA), cloned into the pMD19-T vector (TaKaRa, Dalian, LN, China), and then transformed into competent *E.coli* JM109 cells (TaKaRa, Dalian, LN). Random clones were sequenced with the T7 primer using a BigDye Terminator v3.1 cycle sequencing kit (Applied Biosystems, Inc., Foster, CA). All sequences were trimmed to remove the vector regions and low-quality ends using the PREGAP4 program of the STADEN software package [24]. The sequences were then compared to GenBank sequences using Blastx, and the most similar UreC sequences derived from known bacterial species were downloaded and combined with the UreC protein sequences recovered in this study. A phylogenetic tree was constructed from the combined UreC sequences using the MEGA software [25].

### Expression and purification of urease alpha subunit (UreC)

The gene encoding the UreC of *H. pylori* was amplified from the genomic DNA of *H. pylori* UMAB41 using a forward primer (5′-AAAA*CATATG*AAAAAGATTAGCAGGAAAG-3′) with a *Nde*I cutting site (italic) and a reverse primer (5′-CCG*CTCGAG*CTACCGCGCCATCTTCCACCAG-3′) with a *Xho*I cutting site (italic). Following double digestion with *Nde*I and *Xho*I, the purified full-length gene was ligated into correspondingly double-digested pET-30a(+) (Novagen, Madison, WI). The recombinant plasmids were then transformed into competent *E.coli* BL21 (DE3) (Promega, Madison, WI). The transformants were grown until an OD_600_ of 0.6, and over expression of the cloned UreC was induced at 30°C by IPTG (1.0 mM). The *E. coli* cells were harvested by centrifugation, and total cell protein was isolated using the BugBuster® Protein Extraction Reagent (Novagen, Madison, WI). Purification of the overexpressed UreC was achieved using a Ni-NTA kit (Novagen, Madison, WI) per manufacturer’s instructions. The UreC protein was analyzed by SDS-PAGE and visualized after staining with Coomassie Blue R-250. The UreC protein concentration was determined using the Bradford assay (Bio-Rad, Hercules, CA).

### Purification of rumen urease and western blotting using UreC of ***H. pylori***

Rumen fluid was collected fistulated dairy cows fed a total mixed ration (TMR) (see Additional file 3). The bacterial cells were isolated by gradient centrifugation and then disrupted by ultrasonication (300 W, 15 min). The cellular proteins were concentrated using ultrafiltration (50 kDa) and then a Hi Trap Capto Q ion exchange column (GE Healthcare, Little Chalfont, UK) that was pre-equilibrated with a Tris–HCl buffer (20 mm, pH 8.0). Gradient elution was used to separate urease protein using the same Tris–HCl buffer with a NaCl concentration ranging from 0 to 1 M at a flow rate of 1 mL min^−1^. The fractions with positive urease activities were pooled and concentrated by lyophilization [26].

The urease protein was separated on SDS-PAGE and transferred to a nitrocellulose membrane (Sigma, St Louis, MO, USA) for immune blotting analysis. The membrane was blocked with 5% low-fat dry milk dissolved in TBS-T buffer for 1 h at room temperature and then stained overnight with the bovine anti-urease serum (1:1000). Following three washes for 10 min each in TBS-T buffer, the membrane was incubated in diluted (1:1000 diluted) horseradish peroxidase-conjugated sheep anti-bovine antibody (Bethyl Laboratories, Montgomery, TX) for 1 h at room temperature before incubation in 6 mL of chemiluminescence reagent (Sigma, St Louis, MO, USA) for 1 min. Positive immunostaining was determined based on the presence of a visible band corresponding to the expected UreC protein.

### Immunization of dairy cows

Eight rumen-fistulated lactating Chinese Holstein dairy cows, with a body weight of 556 ± 19 kg, were randomly allocated to two treatment groups (n = 4), with one group (control) mock vaccinated with physiological saline containing the Freund’s adjuvant only, while the other group (vaccinated) was vaccinated with the overexpressed UreC of *H. pylori*. Briefly, an UreC protein solution (0.4 mg mL^−1^ UreC protein) was combined with an equal volume of Freund’s complete adjuvant. The mixture was emulsified, resulting in an UreC vaccine containing 0.2 mg mL^−1^ UreC protein. Each cow in the vaccinated group was injected subcutaneously on the neck and intramuscularly on the buttock [27] with 0.5 mL UreC vaccine at day 0. The injections were repeated at days 14, 28 and 42 as boosters, but with the Freund’s complete adjuvant being replaced by Freund’s incomplete adjuvant. The control group received the same injection procedures in parallel but with physiological saline containing Freund’s adjuvant only. All cows were housed under identical conditions and fed the same TMR (see Additional file 3) thrice daily. The animals were strictly cared for following the standard protocols approved specifically for this study by the Institute of Animal Science, Chinese Academy of Agricultural Sciences, Beijing, China (Permit Number: RNL10/08).

### Animal sample collection and analysis

At days 0, 7, 21, 35, and 49, samples of blood, saliva, and rumen fluid were collected about 2 h after morning feeding. Blood samples were collected from the caudal vein of each cow into evacuated tubes and centrifuged at 3000 × *g* for 10 min to separate the serum. Saliva samples were collected from the oral cavity of the cows using a suction tube and then centrifuged at 10000 × *g* for 15 min to collect the supernatant. Rumen fluid samples were collected through rumen fistula and filtered through four layers of cheesecloth. Subsamples of rumen fluid were also collected for analysis of urease activity. At day 56, 60 g urea was infused directly into the rumen of the cows of both groups through the rumen fistula after morning feeding. Rumen fluid was then collected at 0, 1, 2, 4, 6 and 8 h post infusion and analyzed for pH, ammonia concentration, and VFA profile.

The titers of specific anti-urease IgG and IgA in the serum and the saliva samples were determined using a modified ELISA protocol [28]. Briefly, the plates were coated with 100 μL well^−1^ UreC solution (40 μg mL^−1^) and incubated overnight at 4°C. After washing, the plates were blocked with 150 μL well^−1^ of 1% (vol./vol.) chicken serum for 90 min at 37°C. An aliquot of 100 μL well^−1^ serially diluted serum (1:400 to 1:25600 for IgG, 1:500 to 1:32000 for IgA) and saliva (1:640 to 1:10240 for IgG, 1:20 to 1:640 for IgA) from either the vaccinated cows or the cows in the control group was added. Fetal calf serum was used as a negative control. The plates were incubated at 37°C for 2 h. Then, 100 μL donkey anti-bovine IgG or IgA alkaline phosphatase conjugate (Promega, Madison, WI) (1:10000 diluted) was added to each well and incubated at 37°C for 2 h. Following dilution to a final concentration of 1.5 mg mL^−1^ in a buffer containing 1 M diethanolamine and 0.5 mM MgCl_2_, the substrate chromogen tetramethylbenzidine (100 μL) was added to each well. After incubation for 30 min at 37°C, the reaction was terminated by adding 50 μL of 2 N NaOH to each well. The absorbance was recorded at 405 nm using an ELISA plate-reader (Infinite F200; Tecan, Mannedorf Switzerland). The reaction was defined as positive when the absorbance exceeded twice that of the negative control. Antibody titers were expressed as the highest dilution that gave a positive reaction.

Urease activity was determined by measuring the amount of ammonia released from urea [29]. One unit of urease activity was defined as one μmol ammonia released per min per mL rumen fluid or mg microbial protein. Ammonia concentration was determined by the phenol-hypochlorite reaction as described by Weatherburn et al. [30]. Concentrations of VFA were analyzed by gas chromatography (model 6890, Series II; Hewlett Packard Co., Avondale, PA) as described by Mohammed et al. [31].

### Evaluation of the anti-urease antibody on urea hydrolysis in rumen fluid in vitro

The TMR diet, which was the same as that fed to the cows, was weighed into serum bottles (0.25 g bottle^−1^) containing 20 mL McDougall’s buffer, 10 mL of strained fresh rumen fluid, and urea (final concentration of 1 g L^−1^). To each bottle, bovine serum from either the control group or the vaccinated group was added. Two bovine serum concentrations from the vaccinated group was used in two treatments: Trt1 (IgG titer, 1:40000) and Trt2 (IgG titer, 1:80000). Three replicates were used both the control and the anti-urease serum treatments. The bottles were gassed with CO_2_, sealed with rubber stoppers, and incubated in a 39°C shaking water bath. Subsamples were collected at 0, 1, 2, 4, 8 and 12 h post incubation, and the pH was measured immediately. Ammonia concentrations were determined colorimetrically as described above. Urea concentration was determined with the diacetyl monoxime method of Marsh et al. [32]. Rates of urea disappearance and concomitant ammonia N formation were computed as the slope of regression of the natural logarithm of urea and ammonia N concentration, respectively, over the course of the incubation.

### Statistics

All data were subjected to analysis of variance using the MIXED procedure of SAS (version 9.0, SAS Institute Inc., Cary, NC). The REPEATED statement was used for variables measured over days (titers of IgA and IgG, and urease activity) or times (pH, VFA, NH3-N). Tukey multiple comparison test was used to separate the means when significant differences were indicated by the MIXED procedure. Differences were considered significant at *P* < 0.05.

### Nucleotide sequence accession numbers

The *ure*C sequences obtained in this study were deposited in the GenBank database under accession numbers JQ611755 to JQ612071.

## Additional files

Additional file 1:
**SDS-PAGE of UreC cloned from** ***H. pylori*** **and overexpressed in** ***E. coli*** **.** Marker, protein molecular weight marker; lane 1, total protein after induction by IPTG; lane 2, total protein from uninduced *E. coli* cells; lane 3, protein after Ni-NTA purification. Additional file 2:
**Rumen fermentation characteristics after immunization.**
Additional file 3:
**Ingredient and chemical composition of the TMR diet.**
