# Supplementary figures and images for: Reducing microbial ureolytic activity in the rumen by immunization against urease therein
Source: BMC Vet Res. 2015 Apr 14;11:94. doi: 10.1186/s12917-015-0409-6 (PMC4404106; doi:10.1186/s12917-015-0409-6)

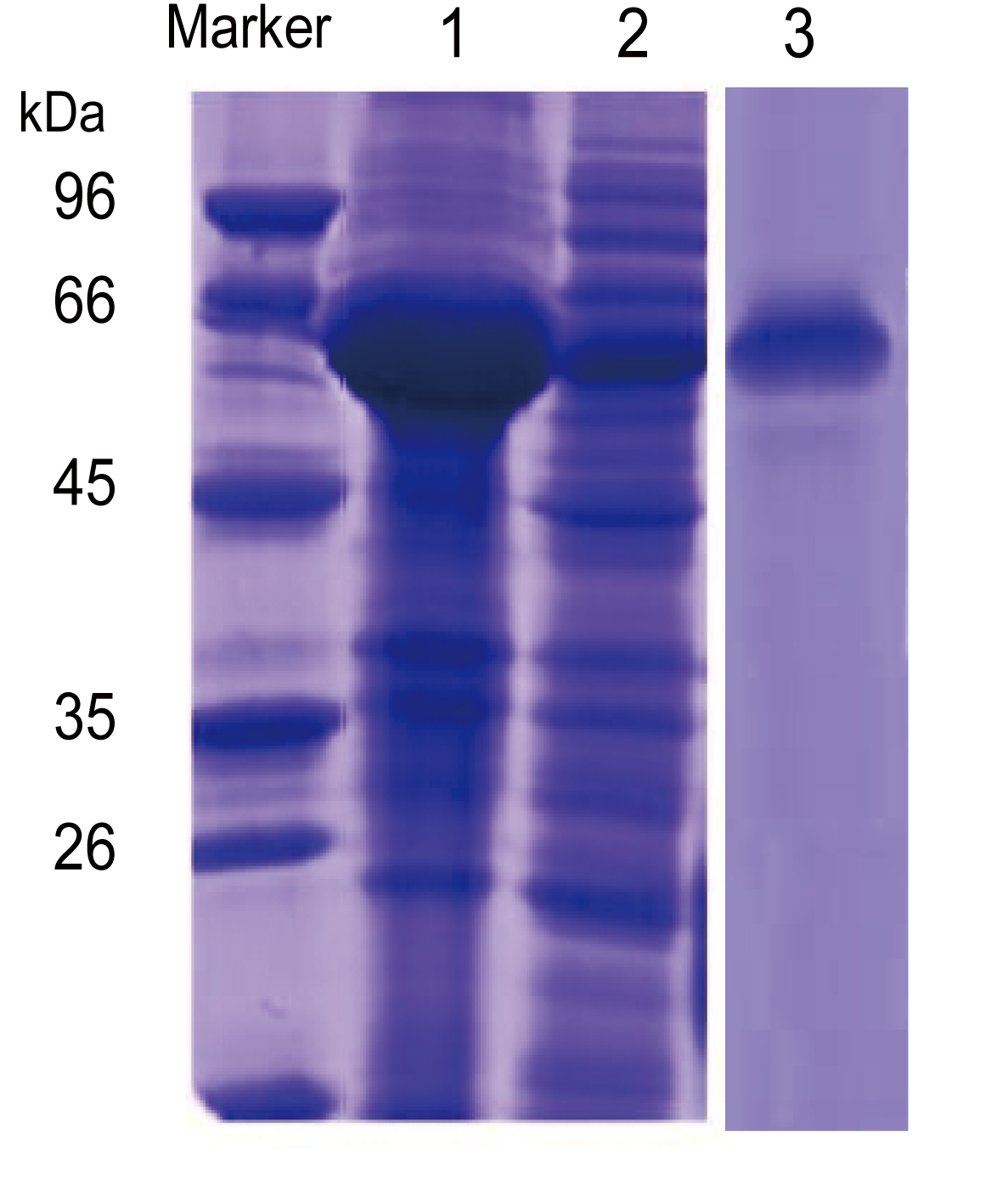

Supplement: Additional file 1: — SDS-PAGE of UreC cloned from H. pylori and overexpressed in E. coli . Marker, protein molecular weight marker; lane 1, total protein after induction by IPTG; lane 2, total protein from uninduced E. coli cells; lane 3, protein after Ni-NTA purification. [file 12917_2015_409_MOESM1_ESM.tiff]
