# Supplementary material for: Reducing microbial ureolytic activity in the rumen by immunization against urease therein
Source: BMC Vet Res. 2015 Apr 14;11:94. doi: 10.1186/s12917-015-0409-6 (PMC4404106; doi:10.1186/s12917-015-0409-6)
Supplement: Additional file 2: — Rumen fermentation characteristics after immunization. [file 12917_2015_409_MOESM2_ESM.docx]

**Additional file 2. Rumen fermentation after immunization.**

| Measurements | Treatments | | SEM | *P* values | | |
| --- | --- | --- | --- | --- | --- | --- |
|  | Control | Vaccinated |  | Treatment | Time | Treatment x Time |
| pH | 6.38 | 6.40 | 0.08 | 0.88 | 0.04 | 0.09 |
| Total VFA, mM | 98.80 | 108.97 | 8.45 | 0.25 | 0.31 | 0.64 |
| Acetate, mM | 67.73 | 74.41 | 5.05 | 0.19 | 0.31 | 0.75 |
| Propionate, mM | 15.81 | 17.05 | 1.76 | 0.52 | 0.14 | 0.16 |
| Butyrate, mM | 1.00 | 0.98 | 0.11 | 0.32 | 0.20 | 0.47 |
| Isobutyrate, mM | 11.97 | 13.57 | 1.32 | 0.59 | 0.32 | 0.26 |
| Valerate, mM | 1.73 | 1.65 | 0.26 | 0.52 | 0.43 | 0.25 |
| Isovalerate, mM | 1.16 | 1.32 | 0.19 | 0.99 | 0.99 | 0.43 |
| Acetate : propionate ratio | 4.21 | 4.41 | 0.20 | 0.52 | 0.05 | 0.05 |
