# Supplementary material for: Reducing microbial ureolytic activity in the rumen by immunization against urease therein
Source: BMC Vet Res. 2015 Apr 14;11:94. doi: 10.1186/s12917-015-0409-6 (PMC4404106; doi:10.1186/s12917-015-0409-6)
Supplement: Additional file 3: — Ingredient and chemical composition of the TMR diet. [file 12917_2015_409_MOESM3_ESM.docx]

Additional file 3. Ingredient and chemical composition of the diet

| Items | % of DM |
| --- | --- |
| Ingredient |  |
| Alfalfa hay | 7.0 |
| Chinese wildrye | 22.0 |
| Corn silage | 21.0 |
| Corn | 23.0 |
| Wheat bran | 3.5 |
| Soybean meal | 10.5 |
| Cotton seed meal | 5.5 |
| Rapeseed meal | 4.0 |
| Calcium carbonate | 1.2 |
| Calcium phosphate ,dibasic | 1.3 |
| Salt | 0.5 |
| Mineral-vitamin premix^*^ | 0.5 |
| Chemical composition |  |
| DM, % | 51.9 |
| CP, %DM | 16.9 |
| NDF, %DM | 37.42 |
| ADF, %DM | 22.43 |
| EE, %DM | 3.09 |
| Ca, %DM | 0.95 |
| P, %DM | 0.64 |

^*^Mineral-vitamin premix: Fe 5.5 mg/g, Cu 4.08 mg/g, Zn 17.5 mg/g, Mn 4.98 mg/g, Se 0.11 mg/g, I 0.18 mg/g, Co 0.088 mg/g, vitamin A 2000 IU/g, vitamin D_3_ 600 IU/g, vitamin E 10.8 mg/g.
